# Poly(A)+ Sensing of Hybridization-Sensitive Fluorescent Oligonucleotide Probe Characterized by Fluorescence Correlation Methods

**DOI:** 10.3390/ijms22126433

**Published:** 2021-06-16

**Authors:** Bjorn Paulson, Yeonhee Shin, Akimitsu Okamoto, Yeon-Mok Oh, Jun Ki Kim, Chan-Gi Pack

**Affiliations:** 1 Asan Institute for Life Science, Asan Medical Center, Seoul 05505, Korea; bjorn.paulson+mtrls@gmail.com; 2 Department of Convergence Medicine, University of Ulsan College of Medicine, Seoul 05505, Korea; dusgo147@mail.ulsan.ac.kr; 3 Research Center for Advanced Science and Technology, The University of Tokyo, 4-6-1 Komaba, Meguro-ku, Tokyo 153-8904, Japan; okamoto@chembio.t.u-tokyo.ac.jp; 4 Department of Pulmonary and Critical Care Medicine, University of Ulsan College of Medicine, Seoul 05505, Korea; ymoh55@amc.seoul.kr

**Keywords:** exciton-controlled hybridization-sensitive oligonucleotide probe, fluorescence correlation spectroscopy, dual-color fluorescence cross-correlation spectroscopy, poly(A) tail, mRNA

## Abstract

Ribonucleic acid (RNA) plays an important role in many cellular processes. Thus, visualizing and quantifying the molecular dynamics of RNA directly in living cells is essential to uncovering their role in RNA metabolism. Among the wide variety of fluorescent probes available for RNA visualization, exciton-controlled hybridization-sensitive fluorescent oligonucleotide (ECHO) probes are useful because of their low fluorescence background. In this study, we apply fluorescence correlation methods to ECHO probes targeting the poly(A) tail of mRNA. In this way, we demonstrate not only the visualization but also the quantification of the interaction between the probe and the target, as well as of the change in the fluorescence brightness and the diffusion coefficient caused by the binding. In particular, the uptake of ECHO probes to detect mRNA is demonstrated in HeLa cells. These results are expected to provide new insights that help us better understand the metabolism of intracellular mRNA.

## 1. Introduction

Localizing ribonucleic acids (RNAs) and determining their intracellular dynamics are longstanding challenges in biochemistry and cell biology [1] and are critical for understanding a wide variety of cellular activities [2]. Of particular interest are the synthesis, folding, modification, processing, and degradation of messenger RNA (mRNA), the expression of which is in turn mediated by the dynamics of microRNAs (miRNAs) [3]. While the transport of amino acids is performed by ribosomal RNA (rRNA) [4], numerous non-coding RNAs process rRNA and methylate deoxyribonucleic acid (DNA), synthesize telomeres, and modulate protein function [5]. For example, transfer RNAs (tRNAs) and small nuclear RNAs (snRNAs) catalyze protein synthesis [6] and mRNA splicing [5], respectively. Thus, there is significant interest in applying the high spatial and temporal resolution of optical microscopy to localizing and determining the dynamics of intracellular RNA.

Determining the localization, expression, kinetics, and function of mRNA in live cells has significance for the understanding of gene expression and gene regulation. As mRNAs have no distinguishing features in unstained brightfield microscopy, several techniques have been developed for the in vitro imaging of mRNAs in fluorescent microscopes. Each of these methods has its strengths and drawbacks. The traditional method of fluorescence in situ hybridization (FISH) requires cells to be fixed and washed to remove nonspecific fluorescence, precluding the visualization of live RNA dynamics. As a result, live-cell fluorescent methods based on oligonucleotides with bound fluorescent labels have been developed. In the commonly used MS2-green fluorescent protein (GFP) system, mRNA is tagged via the genetic incorporation of RNA stem loops that bind the fluorescent proteins [1,7,8]. Numerous derivative probes and improvements to the original MS2-GFP system have been proposed, including reporters with shorter and more configurable RNA binding domains [9,10], repeating strings of fluorescent proteins for increased brightness [10], and split protein complexes that fluoresce conditionally in the case of correct RNA hybridization [11]. FISH has also been recently adapted via endogenous probes for use on live cells [12,13]. However, all these methods require the insertion of reporter genes into the target cell or organism.

As an alternative, the treatment of cells with exogenous RNA-targeting probes can be used to reveal RNA dynamics, as has been demonstrated by the injection of labeled mRNA into oligodendrocytes [14]. Similar probes for tracking RNA in living cells include functionalized quantum dots, which provide high photostability and brightness [7,15,16]; nucleic acid stains [7,17]; probes based on colloidal gold [7,18] and silica [19] nanoparticles; and synthesized DNA-based probes, such as molecular beacons [20,21]. The common methods for the transfection of these probes include microinjection [20], electroporation, and reagent-based methods.

However, many of these probes have low fluorescence specificity for RNA sequences, resulting in a high fluorescence background, and often, several probes are required to bind to a single RNA molecule and obtain consistently observable fluorescence. More recently-developed fluorescent probes for RNA visualization solve the problem of background fluorescence by using RNA hybridization-specific fluorescence. Exciton-controlled hybridization-sensitive oligonucleotide (ECHO) probes exploit the self-quenching, noncovalent dimers of fluorescent dyes that dissociate during hybridization to allow strong RNA-tagged/targeted fluorescence and re-form quenching dimers upon dehybridization to achieve a low fluorescence background [1,22]. These “turn-on probes” have been further enhanced using locked nucleic acids (LNA) for increased stability and single nucleotide polymorphism (SNP)-targetable fluorescence [23]. They have also taken inspiration from miRNA, hybridizing to the poly(A) tail of mRNA [22,24,25].

In almost all eukaryotic cells, complete mRNA are stabilized and prepared for intracellular transport by polyadenylation, which includes the addition of a poly(A) tail to the 3′ end of the RNA molecule. In addition to providing protection against degradation, polyadenylation enables mRNA diffusion/transport from the nucleus via ribosomes for protein synthesis. Transient polyadenylation has also been associated with targeted degradation of cytoplasmic RNA [26], indicating that the intracellular expression of poly(A) is worthy of further scrutiny.

Most of the previous studies applying DNA-based probes, such as the ECHO probe, have focused on single-particle visualization and localization of intracellular mRNA, and there have been few quantitative studies on the dynamic properties of mRNA. While single-molecule detection using GFP or cyanine dye probes has been applied to observe the processes underlying the mRNA metabolism, including the modulation of mRNA decay by promoter proteins [27], it may be of interest to introduce a small-molecule system into cells that allows for the quantification of mRNA dynamics, such as ECHO. As it is still unclear how mRNAs spread throughout individual cells, knowledge of probe dynamics would facilitate quantitative determination of the intracellular kinetic properties of the target mRNA.

Fluorescence correlation spectroscopy (FCS) and dual-color fluorescence cross-correlation spectroscopy (FCCS) are highly sensitive methods for determining the molecular diffusion dynamics and interactions of probe-bound moieties in both aqueous solution and live cells. Based on random fluctuations in fluorescence from a small detection volume as fluorophores are quenched, relaxed, or emitted or transit the volume in question, a correlation function experimentally obtained by FCS can be fit based on kinematic models to determine the state transition parameters or diffusion coefficients (see also Section 4. Materials and Methods). In comparison with other conventional methods, FCS is also robust against absolute concentration and fluidic viscosity, which both. have high local variance in living cells [28,29]. FCS is particularly useful for tracking the diffusional motion of nanoparticles taken up into cells by endocytosis [19,30,31,32,33,34]. Shin et al. have previously used FCS methods to characterize the dark-state and triplet relaxation time of ECHO probes in terms of a two-state excitation model [35]. However, the probes’ luminescence, diffusion coefficients, and kinetic properties have not been previously determined in the cellular environment. Of particular interest are their intracellular behaviors and multi-channel fluorescence capabilities. Furthermore, multi-fluorophore fluorescence, which enables dual-color FCCS, can be useful because it enables enhanced sequence specificity (i.e., molecular interaction) and higher fluorescence signals. Thus, FCCS is promising for detecting the molecular dynamics and kinetics of RNA-bound fluorescence probes in live cells and aqueous solutions.

In this study, thiazole-orange-based ECHO fluorescence probes with different excitation wavelength (D*_nnn_*) designs were combined with FCS and FCCS analyses to achieve insight into the changes in the probes’ fluorescence and diffusion dynamics, both in medium and within cells [22]. The fluorescence intensity of all five D*_nnn_* probes was shown to increase markedly with poly(A) hybridization, with the increases ranging from 1.5× to 12× and with the highest hybridization fluorescence gain occurring for D_514_. Based on the FCS analyses in solution, the diffusion was characterized by a two-component diffusion model, with fast diffusion coefficients around 120 µm^2^/s corresponding to free D*_nnn_* probes and slow coefficients around 9 µm^2^/s corresponding to D*_nnn_* complexes with a target. The dual-colored fluorescence was quantified by FCCS using a one- or two-component diffusion model. An increasing brightness and a dependence on poly(A) concentration were revealed as mixes of two different D*_nnn_* probes bound to poly(A). Based on the evidence of poly(A) binding, it can be concluded that the probes were transiently transfected into cells by electroporation and by using a reagent. The strong fluorescence intensities persisted in the cells, allowing for intracellular comparison of diffusion and transfection behaviors between poly(A) tails under different transfection methods. The measured diffusion coefficients were in good agreement with the results of previous studies on intracellular diffusion and contrasted sharply with those using a transfected non-interfering GFP. Overall, the results indicate that the application of the ECHO probe to an analysis of intracellular mRNA dynamics and metabolism is a promising approach when combined with fluorescence correlation methods.

## 2. Results

**Figure 1:**
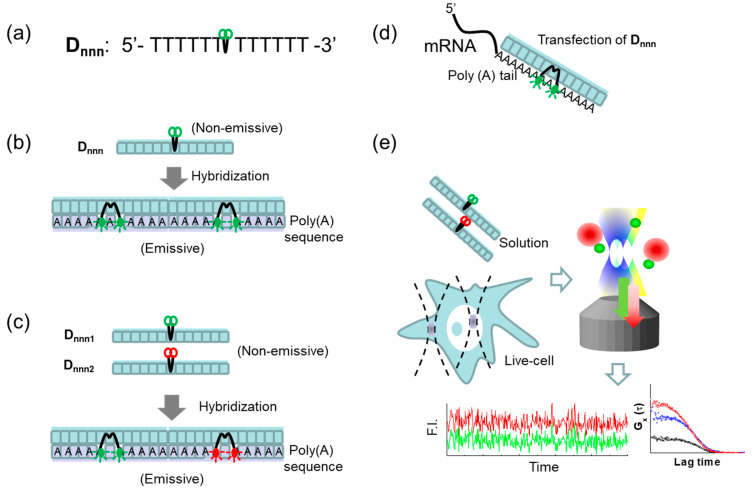
Schematic diagram of hybridization-sensitive fluorescent DNA (D*_nnn_*) probes selectively sensing a poly(A) sequence and the detection of D*_nnn_* by fluorescence correlation methods. (**a**) The structure of the D*_nnn_* probe molecules, consisting of 12 thymine sequences and 2 organic dye molecules (green). The subscript *nnn* denotes the excitation wavelength in nm. (**b**) Schematic representation of a single-color D*_nnn_* probe, detected by FCS, before and after hybridization with the target poly(A) sequence. (**c**) Schematic representation of dual-color D*_nnn_* probes monitored by dual-color FCCS before and after simultaneous hybridization with the target sequence. (**d**) Schematic representation of D*_nnn_* probe hybridized with poly(A) tail of mRNAs in live cell after reagent transfection or electroporation to live cells. (**e**) Schematic representation of FCS and FCCS measurements of sample in aqueous solution and in a live cell after transfection of D*_nnn_* probe. F.I. stands for averaged fluorescence intensity, presented in counts per second (CPS; or kHz). *G_x_*(*τ*) denotes the two fluorescence auto-correlation functions (blue and red) and the cross-correlation function (black; see also Section 4. Materials and Methods).

A series of hybridizing ECHO probes D*_nnn_* were synthesized by a conventional phosphoramidite method [36]. When in the non-hybridized state, these probes undergo fluorescence quenching due to excitonic interactions. As described in detail in a previous paper [22], each fluorescence probe was designed for fluorescence excitation at a specific wavelength *nnn* (in nm). These probes become significantly more fluorescent when hybridizing into DNA and RNA, as the intercalation of the dye into the nucleic acid structure separates the dimers and deactivates quenching via non-emissive transitions [37]. For this study, D*_nnn_* fluorophores were incorporated into poly(T) chains, as shown in Figure 1a, to enable hybridization to targets, such as the poly(A) sequence and poly(A) tail of mRNA. The 5′-d(T_6_D*_nnn_*T_6_)-3′ poly(T) chain is long enough to be specific for poly(A) while also being short enough to allow multiple particles to hybridize to individual poly(A) targets. In human cells, mRNA poly(A) tails tend to be between 250 and 300 adenines in length [38], allowing for the hybridization of multiple fluorescent probes to the same tail. The schematics of the probe, the schematic hybridization of a single-color fluorescent probe, and simultaneous multi-color ECHO hybridization are illustrated in Figure 1a–c, respectively. When transfected using a reagent or electroporated into the HeLa cells, the 5′-d(T_6_D*_nnn_*T_6_)-3′-tagged mRNA fluoresces from the nucleus and cytosol, allowing for the detection and tracking of mRNA dynamics by confocal microscopy and fluorescence correlation methods (Figure 1d,e).

### Fluorescence Behavior of D_nnn_ before and after Hybridization with Poly(A) Oligomer

**Figure 2:**
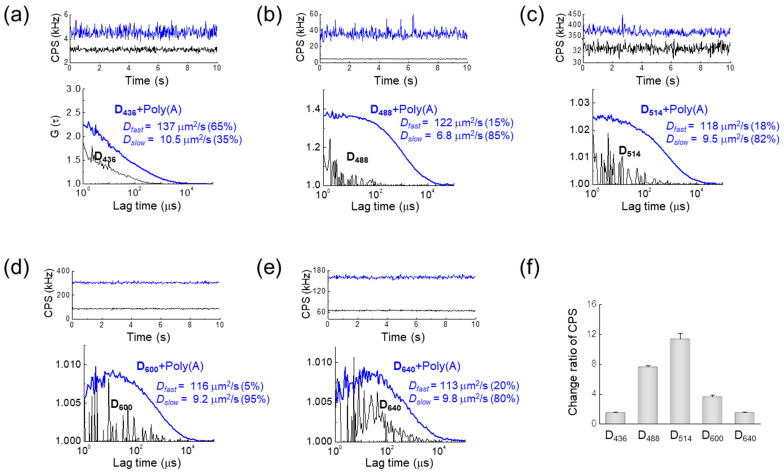
Detection of DNA probe hybridization with poly(A) oligomers by fluorescence correlation spectroscopy for the (**a**) D_436_, (**b**) D_488_, (**c**) D_514_, (**d**) D_600_, and (**e**) D_640_ probes. The subscripts indicate the principal excitation wavelength (nm). (top) Representative averaged fluorescence intensity traces (CPS; kHz) for D*_nnn_* probes alone (black) and hybridized to poly(A) oligomer (blue). (bottom) Fluorescence correlation function *G*(*τ*) between the different dyes before (black) and after (blue) hybridization. The mean diffusion coefficients (D*_fast_* and D*_slow_*) of the free D*_nnn_* probe and hybridized D*_nnn_* complex with poly(A) oligomer are also shown (inset). (**f**) Ratio of averaged fluorescence intensity after and before hybridization for each D*_nnn_* probe.

Although the fluorescence deactivation of the D*_nnn_* probes via dehybridiziation has previously been demonstrated, and their absorption and fluorescence spectra in water are well known [37], their diffusion characteristics and hydrodynamic properties before and after hybridization have not been previously measured in aqueous solution. Figure 2a–e show the FCS evaluations of DNA probes at five different wavelengths before and after hybridization with poly(A) oligomer. All the probes demonstrated significant increases in average fluorescence intensity (CPS) after hybridization (Figure 2f), which is in good agreement with the result of a previous study [36]. While fluorescence intensity was not sufficient to confidently fit non-hybridized D*_nnn_* probe diffusion, except for that of D_436_, the fluorescence correlation functions *G*(*τ*) after hybridization were fit to a two-component model, as described in the Methods section, where the first (“fast”) component captured the effective diffusion coefficient of the free probe molecule in the medium, and the second (“slow”) component captured the effective diffusion due to interactions with poly(A) oligomer. The fast diffusion component was approximately 120 μm^2^/s, corresponding to the values predicted from the molecular weight of the probe (~5 kD), while the slow diffusion components were 10.5, 6.8, 9.5, 9.2 and 9.8 μm^2^/s for the D*_nnn_* complex, including poly(A) oligomer with *nnn* = 436, 488, 514, 600 and 640 nm, respectively. The diffusion coefficients of the complexes formed by each probe were approximately 12 times smaller than that of the free diffusion probe, which corresponds to the diffusion coefficient of linear double-stranded DNA with a size of approximately 300 bp [39,40]. The averaged fluorescence intensity ratios between D*_nnn_* after and before hybridization ranged from 1.52 for D_436_ to 11.37 for D_514_. Although it is unclear why the rate of change of fluorescence intensity varied depending on the type of probe, this may have been caused by the instability of the electrochemical properties of the dye used or the intercalation of the dye to DNA.

**Figure 3:**
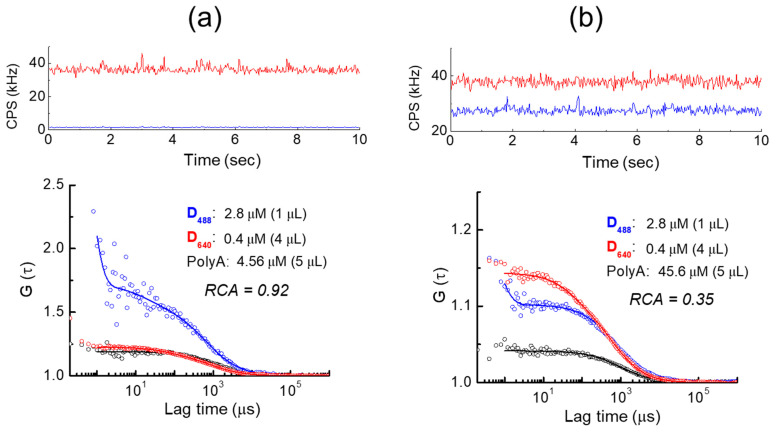
Detection by FCCS of the simultaneous hybridization of two different D*_nnn_* probes with poly(A) oligomers. (**a**) Representative fluorescence intensity traces (top) for a mixture of D_488_ and D_640_ probes with 4.56-μM poly(A), resulting in three fluorescence correlation functions (bottom) and a relative cross-correlation amplitude (RCA) of 0.92. (**b**) Representative fluorescence intensity traces (top) for the same mixture with 45.6-μM poly(A), resulting in fluorescence correlation functions (bottom) with similar diffusion coefficients but an RCA of 0.35. The fluorescence auto-correlation functions of D_488_ and D_640_ are represented in blue and red, respectively. The fluorescence cross-correlation function of the two probes is represented in black.

Following the FCS on single-color D*_nnn_* + poly(A) compounds, dual-color D*_nnn_* mixtures were prepared with D_488_ and D_640_ at a 1.75:1 molar ratio and co-hybridized with sample poly(A) oligonucleotides. As shown in Figure 3, increasing the poly(A) concentration from 4.56 to 45.6 μM greatly decreased the relative fluorescence of D_640_ over D_488_. The fluorescence intensity (CPS) ratio of D_488_ to D_640_ was 0.043:1 at 4.56 μM, whereas it was 0.73:1 with 45.6-μM poly(A). As a result, the relative cross-correlation amplitude (RCA) decreased from 0.92 to 0.35 as the poly(A) concentration was increased, indicating that the number of probes was insufficient due to an increase in the excess poly(A) oligomer. This result demonstrates that using a dual-color probe combined with FCCS makes it possible to detect the binding specifically with the target, which includes gathering information on the binding between the probes as well as on the diffusion coefficient.

**Figure 4:**
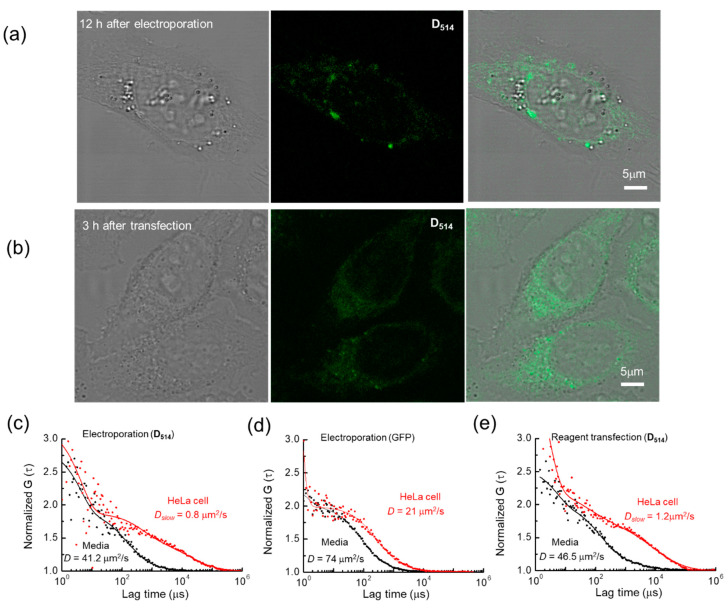
FCS analysis of live HeLa cells transfected with the D_514_ probe. (**a**) HeLa cell after 12 h of electroporation with the D_514_ probe (scale bar: 5 µm). (**b**) HeLa cell after 3 h of transfection with the D_514_ probe (scale bar: 5 µm). (**c**) Representative fluorescence auto-correlation function of the D_514_ probe and (**d**) recombinant GFP molecule in live HeLa cells after electroporation. (**e**) Representative fluorescence auto-correlation functions of the D_514_ probe in live HeLa cells after 3 h of transfection. The black and red curves (solid) are functions fit to the spectra from the culture medium and live cells, respectively. The D value of the D_514_ probe obtained from the fitted curve is also shown (inset). For simplicity, only the diffusion coefficient of the slow component is shown to represent the mobility in the live cells.

In Figure 4, the results of the transient transfection and electroporation of 5′-d(T_6_D_514_T_6_)-3′ into HeLa cells are shown and compared with the electroporation of GFP. A high probe concentration (strong fluorescence) is required for cell imaging using a confocal microscope, whereas FCS analysis is characterized by a high sensitivity at a low concentration (less than 1 μM). Thus, a relatively small concentration of probes was used here compared with that used in most previous studies. As can be seen, 12 h after the electroporation of D_514_ probe into the HeLa cell, the fluorescent probes accumulated into a relatively small number of puncta in the cytoplasm surrounding the nucleus, indicating the agglomeration of probe-tagged mRNA. In contrast, after 3 h of transfection of the D_514_ probe, the fluorescence was widely spread across many smaller fluorescent puncta in the cytoplasm, indicating a relatively uniform uptake throughout the cytoplasm without agglomeration, although any apparent localization may also be partially due to localized enzymatic degradation of the nucleic acid probe [41]. Interestingly, although it should be expected that electroporation will transfer the probe to the cell nucleus, both the electroporation and transfection methods showed that the probe was concentrated more in the cytoplasm than the cell nucleus. Despite their distinct appearances, the diffusion rates of the electroporated and transfected samples were similar, with mean diffusion coefficients between 0.78 and 0.80 μm^2^/s in the cell, respectively (Table 1). In the culture medium, they exhibited similar mean diffusion coefficients of 41.2 and 46.5 μm^2^/s, respectively. These values are 2.5 times lower than the diffusion coefficients of the probe obtained in distilled water, indicating that D_514_ was hybridized to an unknown DNA fragment with the poly(A) sequence contained in the culture medium. In both cases, the electroporated GFP was an order of magnitude more diffusive, with intracellular diffusion coefficients of 21 μm^2^/s in cell and 74 μm^2^/s in culture medium, reflecting the increased viscosity in the cell [42]. The diffusion of D_514_ samples in HeLa cells fit a two-component model, despite the same samples in culture medium fitting a one-component model. In contrast, the diffusion of GFP in both HeLa cells and media solution fit a one-component model [42].

**Figure 5:**
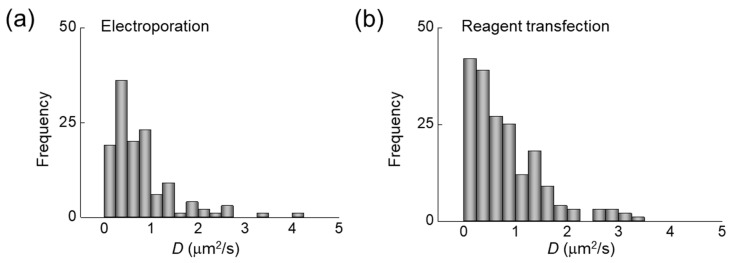
Summary of measured diffusion coefficient D values of the D_514_ probe in live HeLa cells (*n* = 15). The histograms show D** values for the D_514_ probe in HeLa cells after (**a**) electroporation and (**b**) transient transfection using a reagent. For simplicity, only the diffusion coefficient of the slow component is shown.

As shown in Figure 5, a range of diffusion coefficient D values (D*_slow_*) were measured for the D_514_ probe in HeLa cells. The minimum, median, and maximum values of the measured diffusion coefficients were 0.05, 0.57, and 3.3, respectively, in the case of electroporation and 0.04, 0.63 and 3.3, respectively, in the case of reagent transfection. This result demonstrates that the diffusion coefficient of D*_nnn_* introduced into the cell did not differ significantly between the two methods. Importantly, these small diffusion coefficient values correspond to several kbps, considering the high viscosity (increased by approximately 3.5 times) in the cell [42].

## 3. Discussion

It was expected that the precise localization of the synthesized DNA-based fluorescent ECHO probe would be assisted by its low background fluorescence and high fluorescence upon hybridization. Due to the exciton-based quenching between fluorophores, the D*_nnn_* probes exhibited very little fluorescence prior to hybridization. In this study, the fluorescence intensity was enhanced by a factor of between 1.25 and 11.37 upon mixing with a solution of poly(A) ribonucleotide chains. This fluorescence enhancement agrees with the results of prior studies on the binding of ECHO probes to RNA [22,23]. It is also comparable to those of certain probes based on molecular beacon technology, although some molecular beacons have exhibited an order of magnitude more specificity to hybridization [43,44,45]. Expanding on previous studies [35] that determined the band structure of D*_nnn_* and measured its relative diffusion in various buffers and viscosities, the present study aims to establish the diffusion characteristics of D*_nnn_* probes in water and live cells and definitively determine whether mRNA binding is occurring.

The fluorescence correlation function characteristics of poly(A) oligomer were successfully detected from both the single- and double-colored DNA-based D*_nnn_* probes. On the single-colored DNA probe in aqueous solution (i.e., distilled water), FCS generated fluorescence correlation functions that fit well with the two-component diffusion model. The fast diffusion component D*_fast_* represents the diffusion of the free probes in aqueous solution, and its value varied between 113 and 137 µm^2^/s. In contrast, the slow diffusion component D*_slow_* represents the slow diffusion of the hybridized D*_nnn_*-poly(A) complexes in aqueous solution, and its value varied between 6.8 and 10.5 µm^2^/s. Using single-particle tracking, Tadakuma et al. reported a diffusion coefficient of approximately 30 µm^2^/s for mRNA tagged with exogenous enhanced GFP (EGFP) in water along with a diffusion coefficient of 0.2 µm^2^/s for intron-free EGFP-mRNA in the nucleus [46].

Following the FCS evaluation on single-color D*_nnn_* in aqueous solution, dual-color D*_nnn_* mixtures were prepared for FCCS analysis with D_488_ and D_640_ co-hybridized with sample poly(A) oligonucleotides. The RCA between the dual probe channels was observed to decrease from 0.92 to 0.35 as poly(A) oligomer concentration was increased. Although this indicates strong binding to mRNA molecules, these molecules are frequently bound only to a single fluorophore at high poly(A) concentrations. Examinations thus revealed a low cross-correlation between different fluorophores. In contrast, when the poly(A) concentration is low, the binding capacity of the poly(A) saturates, resulting in a great number of poly(A) moieties being host to a mix of probes, which, in turn, have highly correlated fluorescence.

The strong sensitivity of fluorescence to hybridization simplified the assessment of cellular uptake. The uptake by electroporation and reagent transfection was assessed in HeLa cells with the D_514_ probe. When transfected or electroporated into the HeLa cell, the D*_nnn_*-tagged mRNA detected by confocal microscopy was localized to the perinuclear region and the cytosol, allowing the detection and tracking of mRNA dynamics. The electroporated cells expressed punctate fluorescence, with large agglomerations in clumps in the perinuclear region, while the transfected cells expressed fluorescence diffused throughout the cytoplasm. The variance in the patterns of expression was surprising, as the probe was expected to target the poly(A) tails of mRNA, which should be the same in both cases. Although the current images are slightly unclear, electroporated mRNA probes appeared to be highly localized to the cytoplasm. Further examination should be given to determining the cause of this localization, particularly as to whether it arises due to the characteristics of the probe or due to the sensitivity of the intracellular environment to the internalization method. One potential factor in the apparent probe localization is the digestion of the D*_nnn_* probes or hybridized RNA due to localized enzymatic degradation. The intracellular degradation of T6D_535_T6 has previously been determined to result in a 50% reduction in fluorescence over 6 hours [41]. While degradation of the probes or bound RNA may quench fluorescence, affecting the apparent probe localization, probe degradation is not rapid enough to cause measurable error in FCS measurements. In contrast, the cleaving of RNA adjacent to the probe hybridization site could contribute to the measured fast diffusion fraction, but an increase in the fast diffusion fraction is not observed. The measured diffusion coefficients of 0.8 and 1.2 µm^2^/s were slightly higher than those previously documented for the Brownian motion of β-globin and EGFP mRNAs directly injected into the nucleus [46].

The strong fluorescence enhancement upon hybridization with the target sequence and the uptake of the D_514_ probe into the cell offers the opportunity to compare the diffusion rates in the culture medium with those in the cell. The probes in the electroporated HeLa cells exhibited a slow diffusion rate 50 times lower than in medium, while those in the transfected HeLa cells exhibited a diffusion rate 40 times lower. In contrast, when electroporated into HeLa cells alone, the non-hybridizing GFP exhibited a diffusion rate 3.5 times lower than in medium. This provides strong evidence that the diffusion of the probes in the cytosol was not primarily mediated by viscosity and hydrodynamic diameter, as in the case of GFP, but that a significant amount of binding, localization, and perhaps even functional activity of the attached mRNA significantly slowed D_514_ diffusion in the cell. The measured diffusion coefficients were characteristic of mRNA diffusion in cells and were slightly higher than the previously measured diffusion coefficient of 0.2 µm^2^/s for intron-free EGFP-mRNA in the nucleus [46]. The slight discrepancy with this prior study may be due to the size and hydrodynamic diameter of the bound mRNA.

Diffusion methods have previously been used to demonstrate the stability and immobility of D_514_ probes for ECHO-live FISH imaging in HeLa cells. A D_514_-(U)_22_ probe was observed to result in a diffusion coefficient of 0.0004 ± 0.0021 µm^2^/s [13], while D_514_ probes bonded to large nanoparticles have exhibited restricted diffusion across the nuclear membrane [47]. Thus, as a proof-of-concept, this study demonstrates the first measurement of the free diffusion coefficient of the D_514_ probe in the cellular environment.

Taken together, the combination of fluorescence enhancement, dual-color fluorescence, D values, and intracellular dynamics indicate that the D*_nnn_* probes bound strongly to mRNA and tracked their dynamics throughout the cell. While this study demonstrates a proof-of-concept, dual-color method for FCCS via the hybridized fluorescent tagging of RNA, the establishment of these methods is expected to facilitate the visualization and understanding of the function and metabolism of intracellular mRNAs. Further application of these nucleic acid-based probes will be required to discover the ties between the observed dynamics and the full behavior of mRNA in the cellular microenvironment(s).

## 4. Materials and Methods

### 4.1. Cell Culture

A human cancer cell line (HeLa) was obtained from the Korea Cell Line Bank and grown in Dulbecco’s modified Eagle’s medium (DMEM) and fetal bovine serum (FBS; Gibco, ThermoFischer Scientific Korea Ltd., Seoul, Korea). Culturing reagents were purchased from Sigma-Aldrich, St. Louis, MO, USA. Cells were cultured at 37 °C in DMEM containing 10% FBS, 100-U/mL penicillin, and 100-U/mL streptomycin under a humidified 5% CO_2_ atmosphere.

### 4.2. Confocal Laser Scanning Microscopy

Fluorescence confocal microscopy was performed on a LSM510 confocal microscope (Carl Zeiss, Jena, Germany). Illuminations were provided by Ar^+^ ions for a wavelength of 514 nm via a 40× water-immersion objective lens (C-Apochromat, 40×, 1.2 NA; Carl Zeiss). The pinhole size was adjusted to 70 μm.

### 4.3. Fluorescence Correlation Spectroscopy and Fluorescence Cross-Correlation Spectroscopy

The fluorescence correlation analysis has been described in detail in previous papers [35]. Briefly, FCS and FCCS measurements were carried out using a ConfoCor 2 (Carl Zeiss) built on an LSM510 confocal microscope (Carl Zeiss) via a 40× water-immersion objective lens (C-Apochromat, 40×, 1.2 NA; Carl Zeiss). Illuminations were provided by Ar^+^ ions for wavelengths of 458, 488, and 514 nm, and an He–Ne laser for wavelengths of 543 and 633 nm via a 40× water-immersion objective lens (C-Apochromat, 40×, 1.2 NA; Carl Zeiss). Fluorescence was collected using an avalanche photodiode (SPCM-200-PQ; EG&G). Appropriate excitation laser and fluorescent filters were used for each color probe (D_436_: Ex 458 nm, Em 475 nm long-pass filter (LP); D_488_: Ex 488 nm, Em 505 nm LP; D_514_: Ex 514, Em 530−600 nm; D_600_: Ex 543 nm, Em 560 nm LP; D_640_: Ex 633 nm, Em 650 nm LP). The fluorescence correlation functions *G_x_*(*τ*) were calculated using the following equation:

(1) $$G_{x}\left(\tau \right)=1+\frac{\langle \delta I_{i}\left(t\right)\cdot \delta I_{j}\left(t+\tau \right)\rangle }{\langle I_{i}\left(t\right)\rangle \langle I_{j}\left(t\right)\rangle },$$

where *τ* denotes the time delay; *I_i_* denotes the fluorescence intensity of channel *i* (*r* = red, *g* = green); and *G_r_*(*τ*), *G_g_*(*τ*), and *G_c_*(*τ*) denote the auto-correlation function of the red channel (*i* = *j* = *x* = *r*), the auto-correlation function of the green channel (*i* = *j* = *x* = *g*), and the cross-correlation function (*i* = *r*, *j* = *g*, *x* = *c*), respectively.

The *G*(*τ*) values were fit using an *n*-component model, with *n* = 2 being used for the compounds in distilled water and live cells and *n* = 1 being used for the D_514_ probe in culture medium:

(2) $$G_{x}\left(\tau \right)=1+\frac{1}{N}\sum_{i}F_{i}{\left(1+\frac{\tau }{\tau_{i}}\right)}^{-1}{\left(1+\frac{\tau }{s^{2}\tau_{i}}\right)}^{-1/2}.$$

Here, *F_i_* and *τ_i_* are the fraction and diffusion times of component *i,* respectively, *N* is the average number of fluorescent molecules/particles in the detection volume defined by the radius *w*_0_ and the length *2z*_0_, and *s* is the structural parameter representing the ratio *s = z*_0_*/w*_0_. Details on the parameters and the fitting are given in refs. [34,48,49,50].

## 5. Conclusions

This study applied highly sensitive FCS and FCCS analyses to ECHO probes to examine the changes in molecular brightness, diffusion coefficients, and binding affinity with the aim of investigating the binding characteristics of the probe to the target poly(A) sequence. In addition, from an analysis of the probes as detected in live cells, it was possible to indirectly confirm the possibility that the probes were binding to the mRNA through diffusion coefficient analysis. This analysis method will be applicable to various ECHO probes for various RNA targets, and it will be of great help in quantitatively investigating the dynamic properties of intracellular RNA with high sensitivity.

## Figures and Tables

**Table 1:** Comparison of mean diffusion coefficients D of the D_514_ probe and GFP in medium and in HeLa cells.

Fluorescent Probe	D (μm^2^/s)	
	Medium	HeLa
GFP (Electroporation)	74	21
5′-d(T6D_514_T6)-3′ (Electroporation)	41.20	0.78
5′-d(T6D_514_T6)-3′ (Reagent transfection)	46.50	0.80

## Data Availability

Data is contained within the article.

## Abbreviations

CPS	(Fluorescence detection) counts per second
D*_nnn_*	DNA-based ECHO probe of principal excitation wavelength *nnn*
DMEM	Dulbecco’s modified Eagle’s medium
ECHO	Exciton-controlled hybridization-sensitive oligonucleotide
FBS	Fetal bovine serum
FCS	Fluorescence correlation spectroscopy
FCCS	Fluorescence cross-correlation spectroscopy
FI	Averaged fluorescence intensity
FISH	Fluorescence in situ hybridization
GFP	Green fluorescent protein
LNA	Locked nucleic acids
mRNA	Messenger RNA
RCA	Relative cross-correlation amplitude
rRNA	Ribosomal RNA
SNP	Single nucleotide polymorphism
snRNA	Small nuclear RNA
tRNA	Transfer RNA
